# An In-Plane Heterostructure Ni_3_N/MoSe_2_ Loaded on Nitrogen-Doped Reduced Graphene Oxide Enhances the Catalyst Performance for Hydrogen Oxidation Reaction

**DOI:** 10.3390/molecules30030488

**Published:** 2025-01-22

**Authors:** Abrar Qadir, Peng-Peng Guo, Yong-Zhi Su, Kun-Zu Yang, Xin Liu, Ping-Jie Wei, Jin-Gang Liu

**Affiliations:** Key Laboratory for Advanced Materials, School of Chemistry & Molecular Engineering, East China University of Science and Technology, Shanghai 200237, China; abrarqadir67321@gmail.com (A.Q.); 13661917303@163.com (Y.-Z.S.);

**Keywords:** non-precious metal electrocatalyst, hydrogen oxidation reaction, nickel nitride, molybdenum selenide, reduced graphene oxide

## Abstract

Non-noble metal electrocatalysts for the hydrogen oxidation reaction (HOR) that are both highly active and low-cost are essential for the widespread use of fuel cells. Herein, a simple two-step method for creating an in-plane heterostructure of Ni_3_N/MoSe_2_ loaded on N-doped reduced graphene oxide (Ni_3_N/MoSe_2_@N-rGO) as an effective electrocatalyst for the HOR is described. The process involves hydrothermal treatment of the Ni and Mo precursors with N-doped reduced graphene oxide, followed by the annealing with urea. The Ni_3_N/MoSe_2_@N-rGO catalyst exhibits high activities for the HOR, with current densities of 2.15 and 3.06 mA cm^−2^ at 0.5 V vs. the reversible hydrogen electrode (RHE) in H_2_-saturated 0.1 M KOH and 0.1 M HClO_4_ electrolytes, respectively, which is comparable to a commercial 20% Pt/C catalyst under similar experimental conditions. Furthermore, the catalyst demonstrates excellent durability, maintaining its performance during accelerated degradation tests for 5000 cycles. This work offers a practical framework for the designing and preparing of non-precious metal electrocatalysts for the HOR in fuel cells.

## 1. Introduction

The extensive utilization of fossil fuels increases the phenomenon of global warming. It is crucial to identify ecologically sustainable alternatives to address these challenges [1,2,3,4]. Fuel cell technology, which uses hydrogen as a fuel, is considered the most effective and environmentally friendly clean energy source. It has the ability to generate power efficiently without causing pollution. Additionally, it has a wide range of potential applications in sectors such as aerospace, automotive, and portable devices [5,6,7,8,9,10]. Proton exchange membrane fuel cells (PEMFCs) stand out among the various types of fuel cells. In the context of PEMFCs, the advancement of effective and economically viable electrocatalysts is of utmost importance for facilitating both the anodic hydrogen oxidation reaction (HOR) and the cathodic oxygen reduction reaction (ORR) [11,12]. In recent decades, there has been notable advancement in the development of carbon-based catalysts that are not formed of precious metals, with the aim of improving the efficiency of the ORR. Nevertheless, electrocatalysts for the HOR have received significantly less focus [12,13,14,15]. At present, the most common catalysts for the HOR are precious metals, specifically platinum (Pt) and Pt-based alloys. There is a scarcity of information regarding non-precious metal HOR catalysts, with the main emphasis being on nickel-based electrocatalysts. These catalysts have shown a moderate level of electrocatalytic activity, but only when used in alkaline conditions [16,17,18,19,20]. Given that the most common fuel cells, such as PEMFCs, function in extremely acidic conditions, there is a substantial requirement for catalytically active HOR catalysts that exhibit good performance in acidic environments. Thus, a fundamental obstacle persists: creating HOR catalysts that demonstrate superb electrocatalytic performance and great longevity in both acidic and alkaline conditions while substantially decreasing expenses.

Transition metal dichalcogenides (TMDs) are a recently discovered class of two-dimensional materials that exhibit weak van der Waals interaction between layers and covalent bonding of atoms inside each layer, like MoS_2_, MoSe_2_, WSe_2_, and others, demonstrating the development of electrode materials due to their abundant availability, affordable cost, and superior electrocatalytic performance [21,22,23]. Meanwhile, nickel (Ni) compounds have been regarded as favorable catalysts for the HOR in alkaline electrolytes. Nevertheless, pure nickel exhibits poor HOR activity due to the high affinity for hydrogen adsorption. Extensive methodologies have been devised to enhance the HOR performance of catalysts based on nickel. Combining nickel with other transition metals, such as nickel–copper [24], nickel–silver [25], nickel–molybdenum [26], nickel–tungsten [27], and ternary alloys (nickel–tungsten–copper [28], cobalt–nickel–molybdenum [29]), has been found to be an effective method for enhancing the HOR activity. Collaborating with non-metal elements, such as introducing N or O into the catalyst nanoparticle or substrate (e.g., Ni(OH)_2_-Ni/C [30], Ni/N-CNT [31], Ni/Ni_3_N [32], Ni@O_i_-Ni [33], and Ni/SC [20]), has been discovered to be advantageous in enhancing the catalyst performance for the HOR. However, the HOR activity of Ni-based catalysts has always been inferior to the state-of-the-art Pt-based catalysts. Improving the HOR performance of Ni-based catalysts remains a challenging task.

The hydrogen binding energy (HBE) is the main measure of how strongly the HOR intermediates and catalysts stick together. It is widely recognized as the major activity descriptor for HOR catalysts [34]. Cong et al. [35], as well as Yao [36] and Zheng [37], suggested that the adsorption strength of hydrogen is the only characteristic that determines the HOR in alkaline environments. In addition to HBE, researchers attribute the enhanced HOR activity to the optimized binding strength of the surface hydroxyl groups of the catalyst. Strmcnik and colleagues [38], as well as Zhao and colleagues [39], proposed that the HOR activity of catalysts is strongly influenced by their ability to adsorb hydroxyl species. They found that higher adsorption of hydroxyl species is advantageous for the HOR process. So, optimizing both hydrogen binding energy and hydroxyl binding energy contributes to the overall increased activity for the HOR.

**Figure 1:**
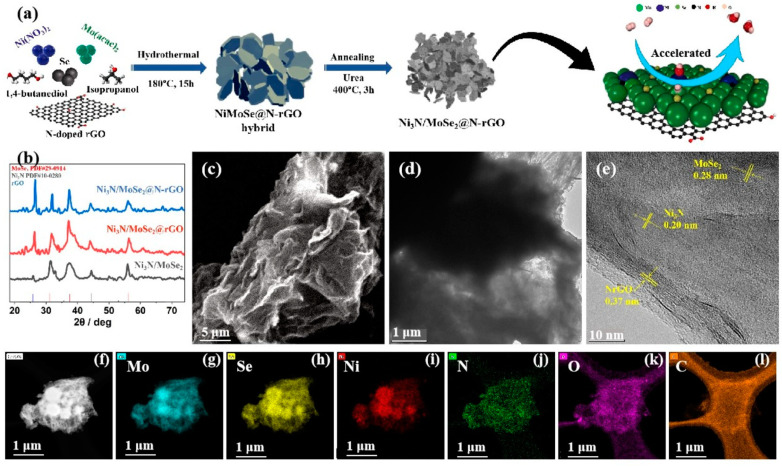
(**a**) Schematic illustration of the preparation of Ni_3_N/MoSe_2_@N-rGO. (**b**) XRD pattern, (**c**) SEM, (**d**) TEM image, (**e**) HR TEM image, and (**f**–**l**) element mapping of Ni_3_N/MoSe_2_@N-rGO.

In this study, we designed Ni_3_N/MoSe_2_ in-plane heterostructures loaded on N-Doped reduced graphene oxide (N-rGO) to improve the HOR activity (Figure 1a). The prepared Ni_3_N/MoSe_2_@N-rGO catalyst demonstrated a high current density of 2.15 mA cm^−2^ and 3.06 mA cm^−2^ and mass activity of 20.4 mA mg^–1^ and 23.92 mA mg^–1^ in alkaline electrolytes and acidic electrolytes, respectively, which is comparable to a commercial 20% Pt/C catalyst under similar experimental conditions. This study presents a practical approach for developing efficient and affordable non-precious metal electrocatalysts for the HOR.

## 2. Results and Discussions

The preparation schedule for obtaining the Ni_3_N/MoSe_2_@N-rGO catalyst is shown in Figure 1a. Firstly, the NiMoSe@N-rGO hybrid was obtained using Mo(acac)_2_, Ni(NO_3_)_2_, and N-rGO as precursors via a hydrothermal treatment, then the hybrid was subsequently annealed with urea at a temperature of 400 °C for 3 h under 5% H_2_/N_2_ atmosphere, which led to the formation of the Ni_3_N/MoSe_2_@N-rGO catalyst composite. The crystal structure and phase composition of the synthesized Ni_3_N/MoSe_2_@N-rGO were determined using XRD analysis, as shown in Figure 1b. The peak at 26.5° indicates the (002) diffraction plane of rGO, the peak at 31.8° represents the (100) lattice plane of MoSe_2_, and the peak at an angle of 37.8° is dedicated to the (103) lattice plane of MoSe_2_ (the sharpening of the peak was due to the increase in the crystallinity of MoSe_2_ by the incorporation of N-rGO). The (111) lattice plane of Ni_3_N is at an angle of 44.0°, and the (110) lattice plane of MoSe_2_ is at an angle of 55.7° (Figure S1) [40,41,42]. The Raman spectrum exhibits a D band at 1349 cm^−1^ and a G band at 1592 cm^−1^ with an I_D_/I_G_ ratio of 1.4 (Figure S2), which indicates the presence of rGO in the composite. The observations validate the successful synthesis of a Ni_3_N/MoSe_2_ in-plane heterostructure loaded on N-doped rGO.

The morphology of in-plane heterostructures of Ni_3_N/MoSe_2_@N-rGO was examined using SEM and TEM. The SEM picture of Ni_3_N/MoSe_2_@N-rGO in-plane heterostructures is depicted in Figure 1c, revealing a sheet-like morphology. The TEM image in Figure 1d provides more information about the structure, showing a sheet-like feature composed of interconnected nanoparticles. Figure 1e displays the high-resolution transmission electron microscopy (HR TEM) picture of Ni_3_N/MoSe_2_@N-rGO. The (111) crystal plane of Ni_3_N has a lattice spacing of 0.20 nm, the (100) crystal plane of MoSe_2_ has a lattice spacing of 0.28 nm, and the (002) crystal plane of N-rGO has a lattice spacing of 0.37 nm. These lattice spacings demonstrate the in-plane heterostructures of Ni_3_N/MoSe_2_@N-rGO. In addition, the elemental mapping study demonstrates the even distribution of the Mo, Ni, N, Se, O, and C elements in the Ni_3_N/MoSe_2_@N-rGO composite (Figure 1f–l).

**Figure 2:**
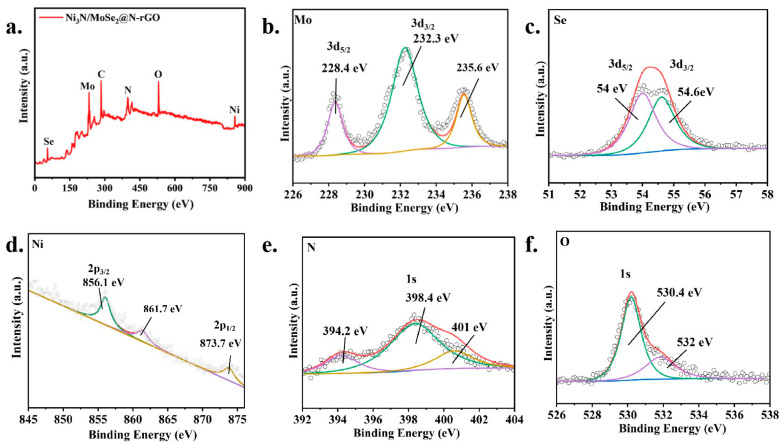
(**a**) Full XPS spectra of Ni_3_N/MoSe_2_@N-rGO, High-resolution (**b**) Mo 3d (**c**) Se 3d (**d**) Ni 2p (**e**) N 1s and (**f**) O 1s XPS spectra of Ni_3_N/MoSe_2_@N-rGO.

The chemical state of the synthesized catalysts was examined using X-ray photoelectron spectroscopy analysis. The survey XPS spectra of Ni_3_N/MoSe_2_@N-rGO revealed that the elements Mo (7.53%), Se (9.49%), Ni (6.95%), N (31.24%), C (28.24%), and O (22.74%) were present in the composite (Figure 2a), consistent with the elemental composition of the samples Ni_3_N/MoSe_2_ and Ni_3_N/MoSe_2_@rGO (Table S1), but they have almost half the percentage of nitrogen compared to Ni_3_N/MoSe_2_@N-rGO, which represents the successful doping of nitrogen in rGO. The high-resolution XPS spectra gave more insights about the elements presented in the composite. The peaks at 228.4 eV and 232.3 eV in the Mo 3d XPS spectra of Ni_3_N/MoSe_2_@N-rGO were assigned to the binding energy of Mo 3d_5/2_ and Mo 3d_3/2_, respectively (Figure 2b). And, the peaks at 54.0 eV and 54.6 eV were attributed to the binding energy of Se 3d_5/2_ and Se 3d_3/2_, respectively (Figure 2c). The peak at 235.6 eV for Mo 3d can be assigned to the unavoidable oxidation of Mo. The peaks at 856.1 eV for Ni 2p_3/2_ and 873.7 eV for Ni 2p_1/2_ were observed in the XPS spectra (Figure 2d). The peak at 861.7 eV for Ni 2p can be assigned to the unavoidable oxidation of Ni. The N 1s XPS spectra of Ni_3_N/MoSe_2_@N-rGO showed three distinct peaks with the binding energy at 394.2 eV, 398.4 eV, and 401.0 eV (Figure 2e). These peaks can provisionally be attributed to N–H bonding, N–Ni, and Mo–N bonding, respectively. The N–H peak is most likely caused by the remaining ammonia that was utilized in the nitridation process [43,44]. The O 1s XPS spectra revealed the presence of peaks at 530.4 eV and 532.0 eV (Figure 2f). Ultimately, the XPS analysis verifies the accomplished formation of Ni_3_N/MoSe_2_@N-rGO [19,45,46,47,48]. From the oxidation states obtained by XPS (Table S1), we can deduce that the partially negative oxidation state of Ni in Ni_3_N suggests that Ni is actively involved in binding hydrogen due to more electron density [49]; the Mo(IV) oxidation state of Mo in MoSe_2_ suggests that Mo might be involved in facilitating the release of protons; the charge transfer between Ni and Mo suggests that the interface between Ni_3_N and MoSe_2_ creates unique active sites with optimized properties for the HOR; pyridinic N in the N-rGO support suggests that these N atoms can act as additional active sites or enhance charge transfer to the Ni_3_N/MoSe_2_ particles, and the presence of unusual peaks indicates the presence of surface defects or vacancies, which can also be active sites for the HOR. The electronic interaction between Ni_3_N and MoSe_2_ in the composite could potentially modify the surface characteristics of the material to optimize the adsorption of *H and *OH and ultimately improve HOR activity.

### 2.1. Electrochemical Evaluation of HOR in Basic Medium

**Figure 3:**
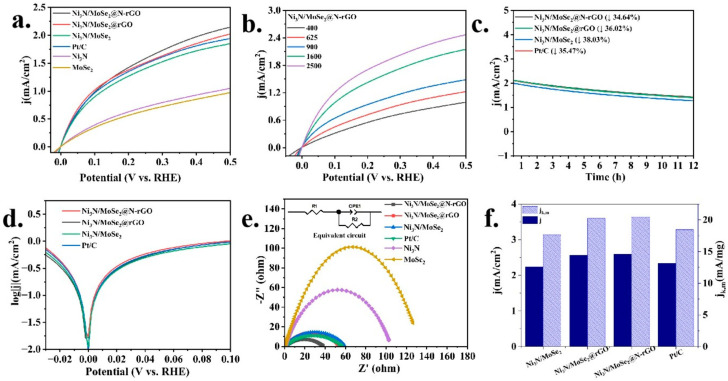
(**a**) HOR polarization curves of Ni_3_N/MoSe_2_@N-rGO, Ni_3_N/MoSe_2_@rGO, Ni_3_N/MoSe_2_, Ni_3_N, MoSe_2_, and commercial Pt/C with a sweep rate of 2 mV s^–1^ in H_2_-saturated 0.1 M KOH. (**b**) polarization curves of Ni_3_N/MoSe_2_@N-rGO at a varied rotation rate, (**c**) chronoamperometric tests at 0.5 V and 1600 rpm, (**d**) Tafel plots, (**e**) EIS spectra, and (**f**) kinetic exchange current density of the catalysts in an H_2_-saturated 0.1 M KOH solution.

The electrocatalytic performance of the catalysts was evaluated in H_2_-saturated 0.1 M KOH electrolytes using a three-electrode setup to measure the HOR. The Ni_3_N/MoSe_2_@N-rGO catalyst exhibited the current density of 2.15 mA/cm^2^ in H_2_-saturated 0.1 M KOH at 0.5 V vs. RHE and the value surpasses those of control samples, Ni_3_N/MoSe_2_ (1.85 mA/cm^2^) and Ni_3_N/MoSe_2_@rGO (2.03 mA/cm^2^) with conventional carbon and rGO as a support, respectively (Figure 3a). Note that the recorded value of 2.15 mA/cm^2^ for Ni_3_N/MoSe_2_@N-rGO exceeded that of the commercial Pt/C (1.96 mA cm^–2^) under similar experimental conditions in 0.1 M KOH, indicating a potential for replacing noble metal catalysts. In addition, the LSV curve of Ni_3_N/MoSe_2_@N-rGO exhibited no significant current during anodic polarization in Ar-saturated 0.1 M KOH (Figure S4). This indicates that the observed current can be attributable to the HOR in H_2_-saturated 0.1 M KOH electrolytes. The polarization curves displayed in Figure 3b demonstrate that the current increases as the rotation rates rise. This is attributed to the enhanced mass transfer at higher rotation rates. The inverse of current densities (j^−1^) was fitted with the square root of the rotation rates (ω^–1/2^). The fitting slope for Ni_3_N/MoSe_2_@N-rGO was found to be 4.71 cm^2^ mA^–1^ s^–1/2^, which is comparable to Pt/C and closely matched the theoretical value of 4.87 cm^2^ mA^–1^ s^–1/2^ for a two-electron HOR process (Figure S5) [50].

The durability of a catalyst over an extended period of time is a critical factor that significantly impacts its practical utilization. Hence, the accelerated durability test (ADT) was performed (at the potential range of −0.1 V to 0.6 V) to assess the stability of Ni_3_N/MoSe_2_@N-rGO. This was achieved by subjecting the catalyst to repetitive CV testing at a scan rate of 100 mV s^–1^. Following 2000 and 5000 CV cycles, the polarization curve exhibited no significant alteration, suggesting the exceptional stability of Ni_3_N/MoSe_2_@N-rGO for the alkaline HOR (Figure S7). In addition, the chronoamperometric tests manifested a current density decline of 34.64% during a period of 12 h in the alkaline medium, which is comparable to that of Pt/C (35.47%, Figure 3c). The Tafel plot, which is derived from the kinetic current, is depicted in Figure 3d. In order to assess the inherent effectiveness of catalysts, the exchange current density (j_0_) can be determined by fitting micropolarization data (Figure S6) [51,52]. The j_0_ value of Ni_3_N/MoSe_2_@N-rGO was determined to be 2.47 mA cm^–2^, which is 3.57 times higher than that of Ni_3_N (0.69 mA cm^–2^) and 4 times higher than MoSe_2_ (0.61 mA cm^–2^) in 0.1 M KOH (Table S2). The observed j_0_ values are compatible with the findings obtained by fitting the Butler–Volmer equation in the Tafel region, as shown in Figure 3d. The kinetic current density (j_k_) was then subsequently computed and standardized by dividing it by the catalyst mass to evaluate the mass activity (j_k,m_). The Ni_3_N/MoSe_2_@N-rGO catalyst displayed mass activity of 20.4 mA mg^–1^ at 0.5 V, which was similar to commercial 20 wt% Pt/C (18.5 mA mg^–1^) in 0.1 M KOH (Figure 3f). To the best of our knowledge, the obtained mass activity was among the top position among the published PGM-free HOR catalysts (Table S3), suggesting that the complex nitride structure placed on N-doped rGO enhances the HOR activity in both alkaline and acidic solutions.

The EIS spectra of the catalysts were recorded. The Ni_3_N/MoSe_2_@N-rGO showed a significantly reduced semicircle in the 0.1 M KOH media, compared to Ni_3_N, MoSe_2_, Ni_3_N/MoSe_2_, and Ni_3_N/MoSe_2_@rGO, when the open-circuit potential (OCP) is applied to the anode of the electrolytic cell (Figure 3e). This indicates that Ni_3_N/MoSe_2_@N-rGO exhibited significantly reduced electric conduction resistance during the electrochemical reaction under OCP conditions. The EIS results were simulated by an equivalent circuit diagram, which is shown in the inset of Figure 3e. In the equivalent circuit diagram of the EIS data, there is a resistor (Rs) connected in series with a resistor (R_ct_) and a constant phase element (C_1_) connected in parallel. R_s_ represents the Ohmic resistance of the electrolyte, while R_ct_ denotes the charge transfer resistance at the interface between the electrolyte and the electrocatalyst. The resistance values (R_ct_) of Ni_3_N/MoSe_2_@N-rGO for the HOR in H_2_-saturated 0.1 M KOH was only 37.9 Ω, which is significantly lower than that of the control samples shown in Figure 3e, suggesting low charge transfer resistance of Ni_3_N/MoSe_2_@N-rGO catalyzed the HOR and thus enhanced HOR activity.

### 2.2. Electrochemical Evaluation of HOR in Acidic Medium

**Figure 4:**
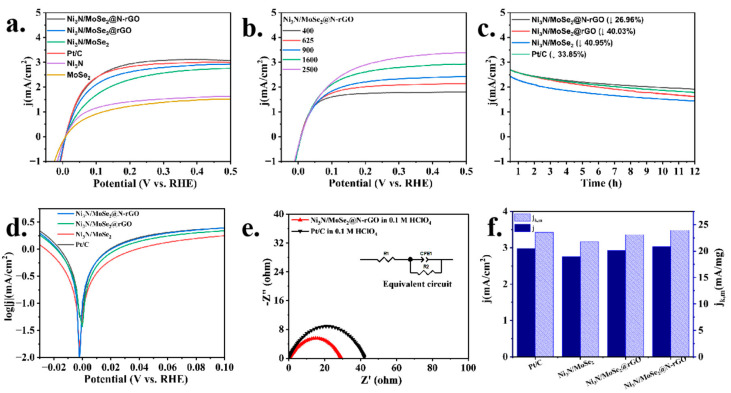
(**a**) HOR polarization curves of Ni_3_N/MoSe_2_@N-rGO, Ni_3_N/MoSe_2_@rGO, Ni_3_N/MoSe_2_, Ni_3_N, MoSe_2_, and commercial Pt/C with a sweep rate of 2 mV s^–1^ in H_2_-saturated 0.1 M HClO_4_. (**b**) polarization curves of Ni_3_N/MoSe_2_@N-rGO at a varied rotation rate, (**c**) chronoamperometric tests at 0.5 V and 1600 rpm, (**d**) Tafel plots, (**e**) EIS spectra, and (**f**) kinetic exchange current density of the catalysts in the H_2_-saturated 0.1 M HClO_4_ solution.

The electrocatalytic performance of the catalysts was also evaluated in H_2_-saturated 0.1 M HClO_4_ electrolytes using a three-electrode setup to measure the HOR. The Ni_3_N/MoSe_2_@N-rGO catalyst showed the current density of 3.06 mA/cm^2^ in H_2_-saturated 0.1 M HClO_4_ at 0.5 V vs. RHE (Figure 4a), which is comparable to that of the commercial Pt/C (3.0 mA cm^–2^) and higher than that of conventional carbon- (Ni_3_N/MoSe_2_, 2.78 mA/cm^2^) and rGO-supported (Ni_3_N/MoSe_2_@rGO, 2.93 mA/cm^2^) samples. The polarization curves in Figure 4b demonstrate that the current increases as the rotation rates rise due to the enhanced mass transfer at higher rotation rates. The polarization curve also exhibited no significant alteration after 2000 and 5000 CV cycles, suggesting good stability of the catalyst in the acidic medium (Figure S8). Furthermore, the Ni_3_N/MoSe_2_@N-rGO catalyst exhibited much-enhanced stability in the chronoamperometric tests, where a much lower current density decrease in Ni_3_N/MoSe_2_@N-rGO (−27%) relative to that of Pt/C (−34%) was noticed during a period of 12 h operation in the acidic medium (Figure 4c). The extended potential range of the linear sweep voltammetry in an acidic medium demonstrated that the Ni_3_N/MoSe_2_@N-rGO catalyst had a higher potential tolerance compared to Pt/C (Figure S9).

The Tafel plot results (Figure 4d) were also consistent with the polarization results shown in Figure 4a. And, the j_0_ value extracted from the fitting of the micropolarization regions [51,52] (Figure S6) was 6.29 mA cm^–2^ for Ni_3_N/MoSe_2_@N-rGO in 0.1 M HClO_4_, which is 1.95 times higher than that of Ni_3_N (3.21 mA cm^–2^) and 2.72 times higher than MoSe_2_ (2.31 mA cm^–2^) (Table S2). The observed j_0_ values are compatible with the findings obtained by fitting the Butler–Volmer equation in the Tafel region, as shown in Figure 4a,f. The Ni_3_N/MoSe_2_@N-rGO catalyst demonstrated a comparable mass activity of 23.92 mA mg^–1^ to that of the commercial 20% Pt/C (23.09 mA mg^–1^) at 0.5 V in 0.1 M HClO_4_ (Figure 4f). When the open-circuit potential (OCP) is applied to the anode of the electrolytic cell, the EIS spectra of Ni_3_N/MoSe_2_@N-rGO showed a reduced semicircle (R_ct_: 29.2 Ω) relative to that of Pt/C in the 0.1 M HClO_4_ electrolyte (Figure 4e), indicating significantly reduced electric conduction resistance for the HOR. The R_ct_ (29.2 Ω) of Ni_3_N/MoSe_2_@N-rGO for the HOR in 0.1 M HClO_4_ is significantly lower than that observed (37.9 Ω) in 0.1 M KOH. This may suggest its higher HOR activity in the acidic electrolyte compared to the alkaline electrolyte.

## 3. Materials and Methods

### 3.1. Chemicals and Reagents

Acetylacetone molybdenum (Mo(acac)_2_), selenium powder and urea were purchased from Sinopharm Chemical Reagent Co., Ltd., Beijing, China. Nickel nitrate (Ni(NO_3_)_2_·6H_2_O), carbon black, ethanol, isopropyl alcohol and 1,4-butanediol were purchased from Shanghai Titan Technology Co., Ltd., Shanghai, China. Potassium hydroxide and Nafion (5%) were purchased from Adamas reagent, Shanghai, China.

#### 3.1.1. Synthesis of NiMoSe Hybrid

Initially, a solution was prepared by dissolving 1.0 mmol of acetylacetone molybdenum (Mo(acac)_2_), 1.0 mmol of Ni(NO_3_)_2_·6H_2_O, and 2 mmol of selenium powder in 35 mL of isopropyl alcohol using ultrasonication. Next, a 10 mL volume of 1,4-butanediol was introduced while applying magnetic stirring for a duration of 1 h, resulting in the formation of a uniform solution. Subsequently, the aforementioned solution was transferred into a Teflon autoclave and maintained at a temperature of 180 °C for a duration of 15 h. Once the NiMoSe hybrid had cooled down to room temperature on its own, it was collected and then washed many times with ethanol. After that, it was dried in a vacuum oven at a temperature of 60 °C overnight.

#### 3.1.2. Synthesis of Ni_3_N/MoSe_2_

The NiMoSe hybrid, manufactured beforehand, was subjected to annealing in a tube furnace. The annealing process involved the addition of 1 g of urea and was carried out at the temperature 400 °C for the duration of 3 h. The annealing was carried out under a 200 sccm flow of a mixture of 5% H_2_/N_2_ gas and a heating rate of 10 °C per minute. The sample that was acquired has been designated as Ni_3_N/MoSe_2_.

#### 3.1.3. Synthesis of Ni_3_N/MoSe_2_@N-rGO

rGO, prepared by the Hummers method [53], and urea were added to water and then subjected to heating at a high temperature and pressure for 10 h in a hydrothermal tank. Then, the precipitates were collected by ultracentrifugation and washed with ethanol and water several times. After drying then overnight, they were annealed with urea at 900 °C temperature for 2 h to obtain N-doped rGO. Ni_3_N/MoSe_2_@N-rGO was then prepared following similar procedures as described for Ni_3_N/MoSe_2_ by adding N-rGO together with the Ni and Mo precursors in the first step of preparing the NiMoSe hybrid.

For comparison, Ni_3_N was synthesized using a similar procedure but without the use of Mo(acac)_2_, and MoSe_2_ was synthesized using a similar method, but without the use of Ni(NO_3_)_2_·6H_2_O, under 5% H_2_/N_2_ and 5% H_2_/Ar atmospheres, respectively.

### 3.2. Physical Characterizations

The images from scanning electron microscopy (SEM) were captured using a Sirion200 field emission scanning electron microscope. The sample morphology was examined using a JEOL JEM1230 transmission electron microscope (JEOL Ltd., Tokyo, Japan) and a high-resolution JEOL JEM1230 transmission electron microscope equipped with an energy-dispersive X-ray spectrometer. X-ray photoelectron spectroscopy (XPS) data were collected using a Thermo Fisher ESCALAB 250Xi spectrometer with a monochromatic Al Kα X-ray source. X-ray diffraction (XRD) analysis was conducted on a Rigaku D/Max 2500 VB2+/PC X-ray powder diffractometer with a monochromatic Cu Kα source, scanning at a rate of 10° per minute. Raman spectroscopy was performed with a Thermo Scientific DXR Raman microscope.

### 3.3. Electrochemical Measurements

The performance of the catalysts for the HOR was investigated using a CHI760D electrochemical workstation that has a three-electrode setup and a rotating disc electrode (RDE). The evaluation was conducted in a 0.1 M KOH and 0.1 M HClO_4_ solution that was saturated with H_2_, respectively. A graphite rod was used as the counter electrode, an Hg/HgO electrode was used as the reference electrode in the alkaline medium, and an Ag/AgCl electrode was used as the reference electrode in the acidic medium. To prepare the working electrode, a mixture of 300 µL of isopropanol and 200 µL of ethanol containing 0.1% Nafion and 2.5 mg of the catalyst (for the sample without support, 1.0 mg of Vulcan XC-72 carbon was also added) was well mixed using ultrasonication. Next, a volume of 10 µL of this ink was administered onto a glassy carbon electrode (pre-polished by alumina slurry), ensuring the catalyst loading of 250 µg/cm^−2^ and left to air-dry, resulting in the formation of the functioning electrode. The working electrode for the control 20 wt% Pt/C (purchased from Johnson Matthey Fuel Cells, Swindon, UK) catalyst was created by dispersing 1.0 mg of the catalyst in 1 mL of isopropanol (0.1% Nafion) and then using ultrasonication to obtain a uniform ink. Afterward, 10 µL of this ink was utilized to create the working electrode, guaranteeing a loading of 10.2 µg_Pt_/cm^2^. The activity of the HOR was assessed via linear sweep voltammetry (LSV) within a voltage range of −0.05 to 0.5 V, employing a scan rate of 2 mV/s and rotation speed of 1600 rpm in an H_2_-saturated 0.1 mol/L KOH and 0.1 M HClO_4_ solution, respectively. All potential values reported have been converted to the RHE scale using the equation E(RHE) = E(vs. Ref) + E°(Ref) + (0.059 × pH).

The Koutecky–Levich equation was employed to estimate the kinetic current density (j_k_) of the catalysts involved in the hydrogen oxidation reaction. The equation may be expressed as follows:

(1) $$\frac{1}{j}=\frac{1}{j_{k}}+\frac{1}{j_{d}}=\frac{1}{j_{k}}+\frac{1}{Bc_{0}\omega^{1/2}}$$

Here j, j_k_, and j_d_ indicate the measured current density, kinetic current density, and diffusion current density, respectively. The Levich constant is denoted by B, the bulk concentration of H_2_ is represented by c_0_, and the rotation speed of the RDE is indicated by ω.

The exchange current density (j_0_) of the HOR catalysts was determined by extracting it from the micropolarization area using linear fitting using the simplified Butler–Volmer Equation (2).

(2) $$j_{0}=jRT/\eta F$$

where R and T represent the universal gas constant and the operating temperature, respectively. The symbols η and F denote the overpotential and the Faraday constant, respectively. Alternatively, j_0_ may also be determined by utilizing the whole Butler–Volmer Equation (3).

(3) $$j_{k}=j_{0}(e^{\left(\alpha F\eta /RT\right)}-e^{(\alpha -1)F\eta /RT})$$

where α represents the charge transfer coefficient, F represents the Faraday constant, and R stands for the universal gas constant. This method enables the assessment of both the mass activity (j_k_) and the overall efficiency (j) of the HOR catalysts.

## 4. Conclusions

To summarize, we have successfully synthesized Ni_3_N/MoSe_2_ in-plane heterostructures loaded on N-rGO using a simple solvothermal–nitridation method. These heterostructures in Ni_3_N/MoSe_2_@N-rGO have proven to be highly effective for the HOR in both acidic and alkaline environments. The prepared Ni_3_N/MoSe_2_@N-rGO catalyst demonstrated comparable HOR activity and superior stability to that of the commercially available Pt/C catalyst in both alkaline and acidic electrolytes. The high HOR performances observed for Ni_3_N/MoSe_2_@N-rGO are attributed to several factors. Firstly, the intimate contact between Ni_3_N and MoSe_2_ facilitates charge transfer at the interface, modulates the electronic structure, and optimizes the adsorption energy of H* and OH*. Additionally, the N-rGO support provides a better surface area, electrical conductivity, and more active sites for the reaction. The optimization of the HBE and OHBE contributes to the improved performance; the support and catalyst work synergistically, promoting the formation of catalytic-active sites that are highly effective in driving the reaction. Due to its simple preparation method, abundant components, and excellent catalytic performance, the Ni_3_N/MoSe_2_@N-rGO electrocatalyst is a very promising substitute for Pt/C in HOR catalysis. This might greatly assist the widespread use of fuel cells on a broad scale.

## Data Availability

Data are contained within the article and supplementary materials.

## Supplementary Materials

The following supporting information can be downloaded at https://www.mdpi.com/article/10.3390/molecules30030488/s1, Figure S1: XRD pattern of Ni_3_N, MoSe_2_, and Ni_3_N/MoSe_2_; Figure S2: Raman spectrum of Ni_3_N/MoSe_2_@N-rGO; Figure S3: SEM image of Ni_3_N/MoSe_2_ sheet; Figure S4: LSV curves of Ni_3_N/MoSe_2_ in-plane heterostructure in H_2_- and Ar-saturated 0.1 M KOH; Figure S5: Fitting slope for Ni_3_N/MoSe_2_, Pt/C, and Ni_3_N/MoSe_2_@N-rGO; Figure S6: micropolarization regions in 0.1 M KOH and 0.1 M HClO_4_; Figure S7: polarization curves of Ni_3_N/MoSe_2_@N-rGO before and after multiple CVs in 0.1 M KOH; Figure S8: polarization curves of Ni_3_N/MoSe_2_@N-rGO before and after multiple CVs in 0.1 M HClO_4_; Figure S9: extended potential LSVs in the acidic medium; Table S1: elemental percentage according to XPS survey; Table S2: exchange current densities of electrocatalysts; Table S3: HOR performances of electrocatalysts in alkaline and acidic medium. References [11,13,16,25,27,30,32,45] are cited in the supplementary material.
