# Supplementary material for: An In-Plane Heterostructure Ni3N/MoSe2 Loaded on Nitrogen-Doped Reduced Graphene Oxide Enhances the Catalyst Performance for Hydrogen Oxidation Reaction
Source: Molecules. 2025 Jan 22;30(3):488. doi: 10.3390/molecules30030488 (PMC11820556; doi:10.3390/molecules30030488)
Supplement: Supplementary file 1 [file molecules-30-00488-s001.zip › molecules-3395596-supplementary (2).pdf]

Supporting Information for

An In-plane Heterostructure $Ni_3N/MoSe_2$ loaded on Nitrogen-Doped Reduced Graphene Oxide Enhances the Catalyst Performance for Hydrogen Oxidation Reaction

Abrar Qadir, Peng-Peng Guo, Yong-Zhi Su, Kun-Zu Yang, Xin Liu, Ping-Jie Wei, and Jin-Gang Liu*

Key Laboratory for Advanced Materials, School of Chemistry & Molecular Engineering, East China University of Science and Technology, Shanghai 200237, P. R. China, E-mail: liujingang@ecust.edu.cn.

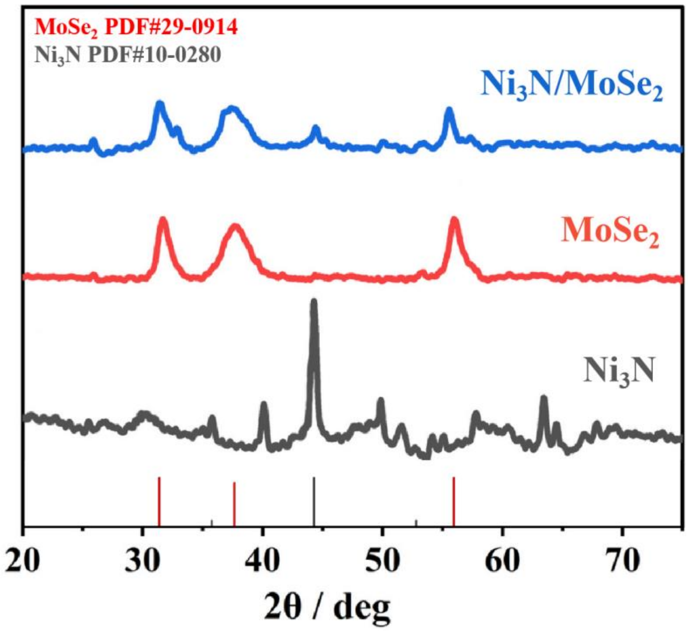

Figure S1. XRD Pattern of Ni_3N, $MoSe_2$ and $Ni_3N/MoSe_2$.

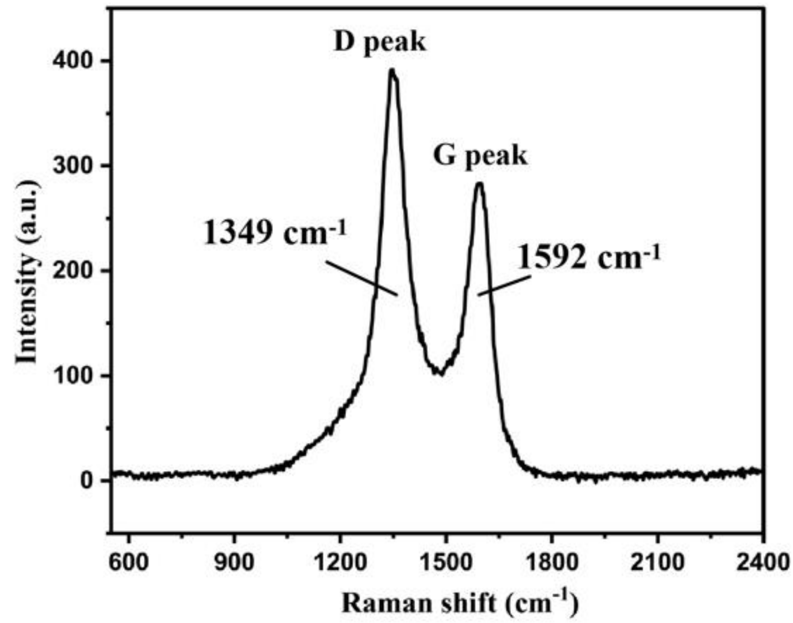

Figure S2. Raman spectrum of $Ni_3N/MoSe_2$@N-rGO.

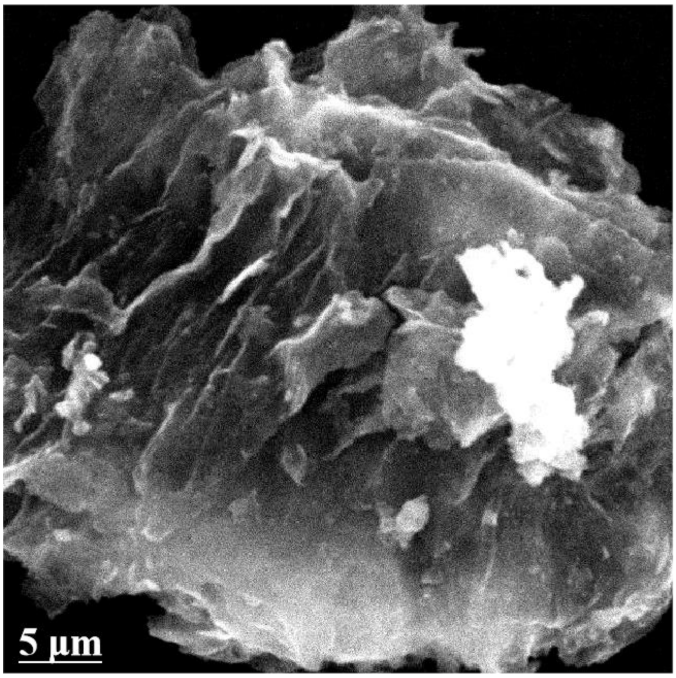

Figure S3. SEM image of $Ni_3N/MoSe_2$ sheet.

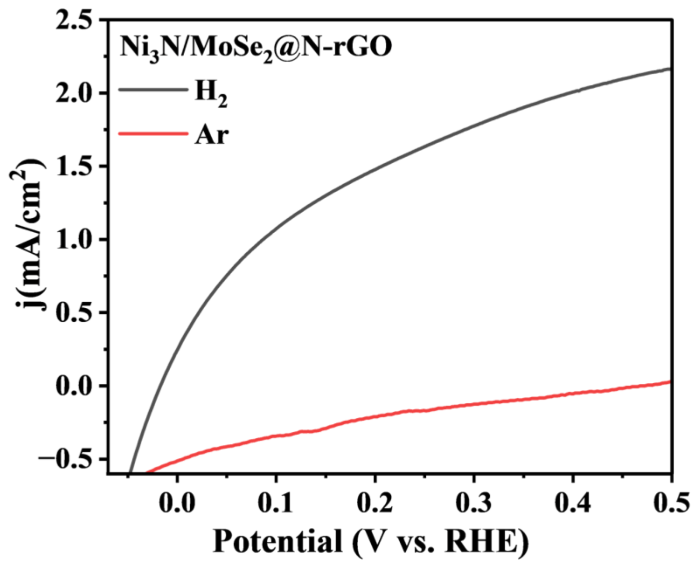

Figure S4. LSV curves of $Ni_3N/MoSe_2$ in-plane heterostructure in H_2 and Ar saturated 0.1 M KOH.

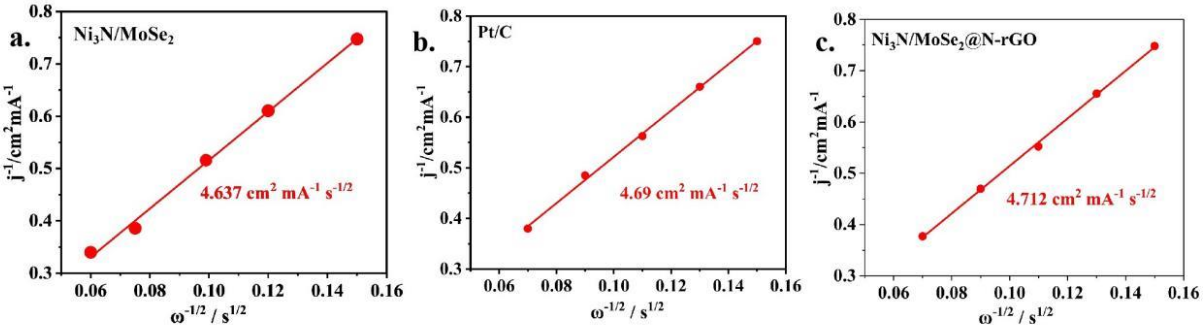

Figure S5. Fitting slope for $Ni_3N/MoSe_2$, Pt/C and $Ni_3N/MoSe_2$@N-rGO.

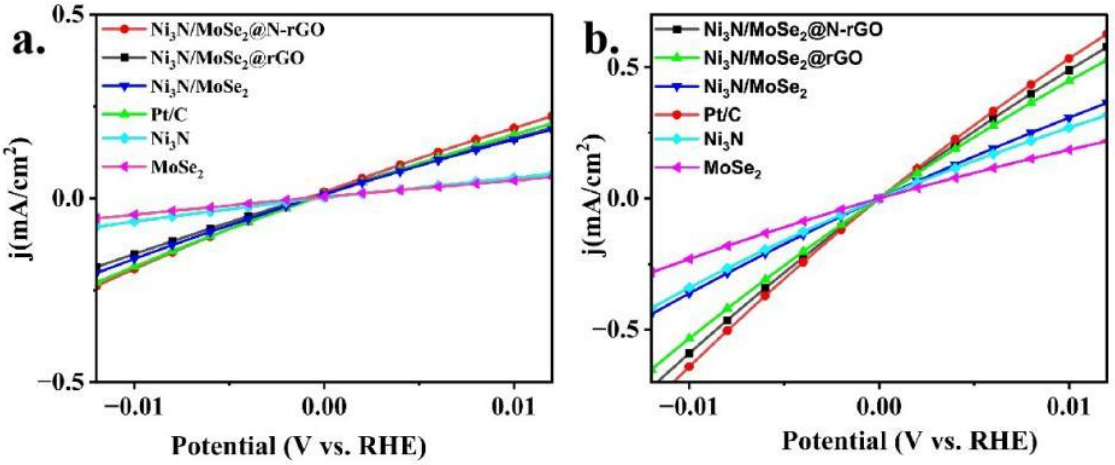

Figure S6. Micropolarization regions in (a) 0.1 M KOH, (b) 0.1 M $HClO_4$.

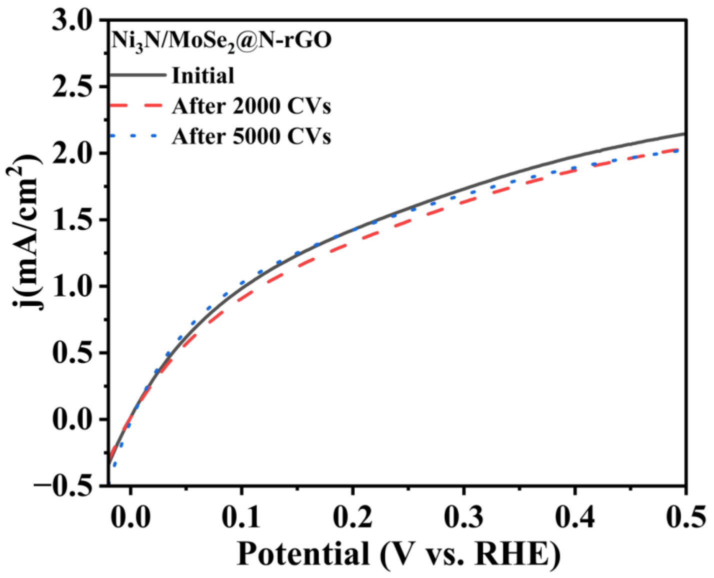

Figure S7. Polarization curves of $Ni_3N/MoSe_2$@N-rGO before and after multiple CVs in 0.1 M KOH.

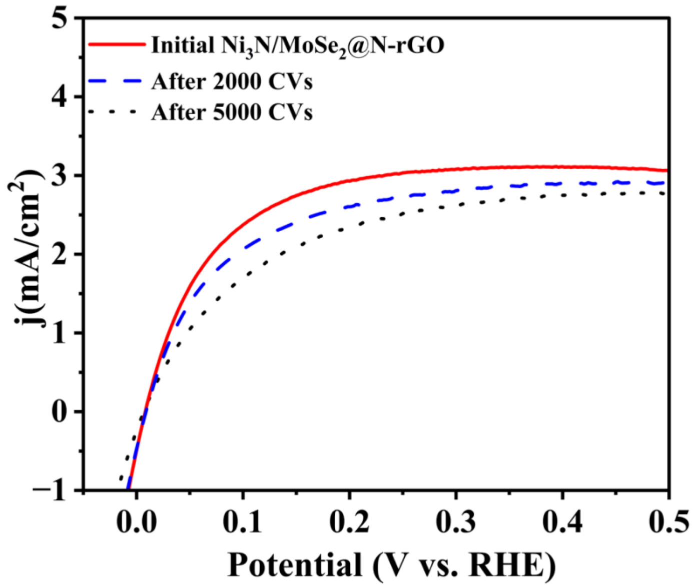

Figure S8. Polarization curves of $Ni_3N/MoSe_2$@N-rGO before and after multiple CVs in 0.1 M $HClO_4$.

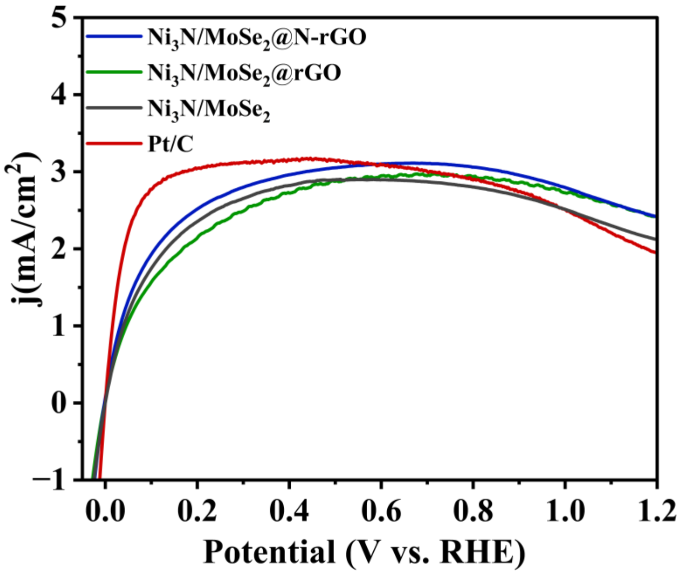

Figure S9. Extended potential LSV's in the acidic medium.

Table S1. Elemental percentage according to XPS survey.

Elements	% in $Ni_3N/MoSe_2$	% in $Ni_3N/MoSe_2$@rGO	% in $Ni_3N/MoSe_2$@N-rGO	Oxidation States
Ni	7.12	7.04	6.95	Ni(δ^-) and Ni(II)
N	16.23	15.85	31.24	
Mo	6.90	7.21	7.53	Mo(IV) and Mo(VI)
Se	8.95	8.73	9.49	
C	-	41.11	28.24	
O	-	10.77	22.74	

Table S2. Exchange current densities of electrocatalysts.

Catalysts	Exchange Current Density (J_0) in 0.1 M KOH (mA/cm^2)	Exchange Current Density (J_0) in 0.1 M $HClO_4$ (mA/cm^2)
$Ni_3N/MoSe_2$@N-rGO	2.47	6.29
$Ni_3N/MoSe_2$@rGO	2.15	5.77
$Ni_3N/MoSe_2$	2.06	3.98
Pt/C	2.23	6.81
Ni_3N	0.69	3.21
$MoSe_2$	0.61	2.31

Table S3. HOR performances of electrocatalysts in alkaline and acidic medium.

Electrocatalyst	Loading capacity (mg cm^{-2})	$j_{0.5V}$ (vs. RHE) (mA cm^{-2})	$j_{k,m}$ (mA/mg)	Electrolytes	Deactivation potential (V)	Ref.
$Ni_3@(h\text{-}BN)_1$	0.42	1.95	16.43	0.1 M NaOH	Up to 0.13	1
Ni/NiO/C-700	0.5	~1	5	0.1 M KOH	Up to 0.10	2
Ni/SC	0.3	~1.15	4.0	0.1 M KOH	Up to 0.10	3
Ni/N-CNT	0.357	~1.32	6.5	0.1 M KOH	Up to 0.08	4
CeO_2(r)-Ni/C	0.306	~1.15	5.67	0.1 M KOH	Up to 0.11	5
hcp/fcc-Ni-C	0.306	~1.7	5.54	0.1 M KOH	Up to 0.10	5
Ni_3N/C	0.31	~1.8	24.38	0.1 M KOH	Up to 0.26	6
Ni/MoO_2	0.77	1.92	12.5	0.1 M KOH	Up to 0.20	7
Ni_4Mo/TiO_2	0.37	–	20.7	0.1 M KOH	Up to 1.2	4
WNPC	0.35	1.03<br>0.91	–	0.1 M $HClO_4$<br>0.1 M KOH	Up to 0.5<br>Up to 0.6	8
*$Ni_3N/MoSe_2$	~0.42	1.85<br>2.78	17.62<br>21.7	0.1 M KOH<br>0.1 M $HClO_4$	1.2<br>Up to 1.12	This work
*$Ni_3N/MoSe_2$ @rGO	–	2.03<br>2.93	20.23<br>23.09	0.1 M KOH<br>0.1 M $HClO_4$	1.0<br>Up to 1.12	This work
*$Ni_3N/MoSe_2$ @N-rGO	–	2.15<br>3.06	20.45<br>23.92	0.1 M KOH<br>0.1 M $HClO_4$	1.0<br>Up to 1.12	This work
*Pt/C	10.2 μg_{Pt} cm^{-2}	1.96<br>3.0	18.41<br>23.5	0.1 M KOH<br>0.1 M $HClO_4$	1.08<br>Up to 0.8	This work

References:

1. Gao, L., Wang, Y., Li, H., Li, Q., Ta, N., Zhuang, L., ... & Bao, X. (2017). A nickel nanocatalyst within a h-BN shell for enhanced hydrogen oxidation reactions. *Chemical science*, *8*(8), 5728-5734.
2. Yang, Y., Sun, X., Han, G., Liu, X., Zhang, X., Sun, Y., ... & Sun, Y. (2019). Enhanced Electrocatalytic Hydrogen Oxidation on Ni/NiO/C Derived from a Nickel-Based Metal–Organic Framework. *Angewandte Chemie International Edition*, *58*(31), 10644-10649.
3. Pedersen, C. M., Escudero-Escribano, M., Velázquez-Palenzuela, A., Christensen, L. H., Chorkendorff, I., & Stephens, I. E. (2015). Benchmarking Pt-based electrocatalysts for low temperature fuel cell reactions with the rotating disk electrode: oxygen reduction and hydrogen oxidation in the presence of CO. *Electrochimica Acta*, *179*, 647-657.
4. Tian, X., Ren, R., Wei, F., Pei, J., Zhuang, Z., Zhuang, L., & Sheng, W. (2024). Metal-support interaction boosts the stability of Ni-based electrocatalysts for alkaline hydrogen oxidation. *Nature communications*, *15*(1), 76.
5. Yang, F., Tian, X., Luo, W., & Feng, L. (2023). Alkaline hydrogen oxidation reaction on Ni-based electrocatalysts: From mechanistic study to material development. *Coordination Chemistry Reviews*, *478*, 214980.
6. Wang, T., Wang, M., Yang, H., Xu, M., Zuo, C., Feng, K., ... & Li, Y. (2019). Weakening hydrogen adsorption on nickel via interstitial nitrogen doping promotes bifunctional hydrogen electrocatalysis in alkaline solution. *Energy & Environmental Science*, *12*(12), 3522-3529.
7. Deng, S., Liu, X., Guo, X., Zhao, T., Lu, Y., Cheng, J., ... & Wang, D. (2021). Insight into the hydrogen oxidation electrocatalytic performance enhancement on Ni via oxophilic regulation of MoO_2. *Journal of Energy Chemistry, 54,* 202-207.
8. Xiong, B., Zhao, W., Chen, L., & Shi, J. (2019). One-Step Synthesis of W_2C@N, P-C Nanocatalysts for Efficient Hydrogen Electrooxidation across the Whole pH Range. *Advanced Functional Materials*, *29*(31), 1902505.
